# Biofilm Formation and Phospholipase and Proteinase Production in *Cryptococcus neoformans* Clinical Isolates and Susceptibility towards Some Bioactive Natural Products

**DOI:** 10.1155/2023/6080489

**Published:** 2023-03-31

**Authors:** Borel Ndezo Bisso, Alvine Lonkeng Makuété, Joël Ulrich Tsopmene, Jean Paul Dzoyem

**Affiliations:** Department of Biochemistry, Faculty of Science, University of Dschang, Dschang, Cameroon

## Abstract

**Background:**

Cryptococcosis is one of the most common fungal infections in immunocompromised patients, which is caused by *Cryptococcus neoformans*. However, relatively little is known about the virulence factors of *C. neoformans* and the incidence of antifungal drug resistance in *C. neoformans* is rapidly increasing. This study was undertaken to investigate the virulence factors in *C. neoformans*, thymol, curcumin, piperine, gallic acid, eugenol, and plumbagin for their potential antimicrobial activity against *C. neoformans*.

**Methods:**

The production of phospholipase and proteinase was detected using standard methods. Biofilm formation was determined using the microtiter plate method. The broth microdilution method was used to determine the antifungal activity. The antibiofilm activity was assessed using the safranin staining method.

**Results:**

All isolates of *C. neoformans* produced biofilms with optical density values ranging from 0.16 to 0.89. A majority of *C. neoformans* isolates that were tested exhibited strong phospholipase (7/8) and proteinase (5/8) production. Plumbagin (with minimum inhibitory concentration values ranging from 4 to 16 *μ*g/mL) showed the highest antifungal activity followed by thymol (with minimum biofilm inhibitory concentration values ranging from 8 to 64 *μ*g/mL). In addition, plumbagin showed the highest antibiofilm activity with minimum biofilm inhibitory concentration and minimum biofilm eradication concentration values ranging from 4 to 16 *μ*g/mL and 32 to 256 *μ*g/mL, respectively.

**Conclusion:**

Plumbagin, compared to other natural products studied, was the most efficient in terms of antifungal and antibiofilm activities. Hence, plumbagin could be used in combination with antifungals for the development of new anticryptococcal drugs.

## 1. Introduction

*Cryptococcus neoformans* is a major opportunistic pathogen causing cryptococcosis. Cryptococcosis occurs when yeast is inhaled into the lungs, which proliferates and disseminates to the central nervous system mostly in individuals with immune system deficiencies leading to meningitis that represents the major cause of death in people living with HIV/AIDS [1]. Approximately, one million cases of cryptococcosis meningitis have been reported in HIV/AIDS patients with over 600 000 deaths per year worldwide [2]. In sub-Saharan Africa, the global annual incidence estimate for cryptococcosis cases exceeds 957 900 with 180 000 deaths [3, 4]. In fact, several virulence factors, such as biofilm formation, and the secretion of phospholipases and proteases contribute to the success of fungal infection [5]. *C. neoformans* grows as a biofilm. Biofilms are communities of aggregated microbial cells embedded in an extracellular matrix composed of water, polysaccharides, lipids, proteins, and extracellular DNA [6]. Biofilm formation confers increased resistance of cells inside biofilms to antimicrobials and hosts immune mechanisms compared with their planktonic cells [7]. The phospholipases hydrolyse the ester linkages on membrane phospholipids, releasing fatty acids as a potential energy source of *C. neoformans* [8]. Proteases can interfere with the host defense mechanisms by cleaving essential immunological components and directly damaging the effector cells [8, 9]. In addition, serine protease secreted by *C. neoformans* promotes increased blood-brain barrier permeability [8, 10].

Therapeutic options for the treatment of cryptococcosis are limited to polyenes, azoles, and 5-flucytosine. However, these classes of antifungal drugs are hampered by host toxicity and pathogen resistance. In addition, the lack of access to drugs and the high cost of effective treatment hamper therapeutic options [11, 12]. Therefore, the development of new therapeutic strategies is urgent.

Natural products from plants can have the potential for the development of new antimicrobials with high activity, low toxicity, and the capacity to enhance the antimicrobial activity of conventional drugs [13]. Thymol is a monoterpene phenol compound that is a main constituent of the essential oils of thymus. Piperine is an alkaloid present in *Piper nigrum* and *Piper longum* vulgaris [14]. Gallic acid, curcumin, eugenol, and plumbagin are natural phenolic compounds found in *Caesalpinia mimosoides*, *Curcuma longa*, *Eugenia caryophyllata,* and *Plumbago zeylanica,* respectively. These natural products have been shown to possess several pharmacological properties such as antioxidant, anticancer, anti-inflammatory, immunomodulatory, antibacterial, antifungal, antibiofilm, and antiviral properties [15–20]. However, up to now, the antifungal and antibiofilm activities of these natural products against *C. neoformans* have remained poorly studied. The present study aimed to investigate the virulence factors in *C. neoformans* and the potential antifungal and antibiofilm activities of thymol, curcumin, piperine, gallic acid, eugenol, and plumbagin against *C. neoformans.*

## 2. Materials and Methods

### 2.1. Microorganisms and Culture Media

A total eight clinical isolates, namely, Cn46, Cn47, Cn91, Cn96, Cn118, Cn158, Cn169, and Cn173, were used in this work. The clinical isolates of *C. neoformans* were obtained from the HIV-positive patients at the Yaoundé Central Hospital and identified by serotyping by multiplex PCR in a previous study [21]. Sabouraud Dextrose agar (SDA, Liofilchem) and Sabouraud Dextrose broth (SDB, Liofilchem) were used for the activation of yeasts and antimicrobial assays, respectively.

### 2.2. Chemicals

Thymol (purity ≥98.5%), curcumin (purity ≥98%), piperine (purity ≥97%), gallic acid (purity ≥97%), eugenol (purity ≥99%), plumbagin (purity >98%), amphotericin B, fluconazole, and dimethyl sulfoxide (DMSO) were purchased from Sigma Aldrich.

### 2.3. Biofilm Formation Assay

The biofilm formation assay was performed using the microtiter plate method as previously described by Bisso et al. with some modifications [6]. Standard cultures (1.5 × 10^6^ CFU/mL) were diluted 100-fold in SDB supplemented with 2% glucose. From each culture, 200 *μ*L of the solution was introduced into a 96-wellflat-bottomed sterile polystyrene plate and incubated at 37°C for 48 h. After incubation, the wells were gently discarded and washed three times with sterile phosphate-buffered saline (PBS, pH 7.2) to remove the planktonic cells. Then, 150 *μ*L of methanol was transferred into each well to fix adherent cells, and the plates were incubated at room temperature for 20 min. After incubation, the wells were discarded and 150 *μ*L of safranin (1%) was added to each well to stain the biofilm. After incubation at room temperature for 20 min, the wells were discarded and 150 *μ*L of 95% ethanol was added to each well to solubilise the dye bound to adherent cells. The optical density (OD) of plates was spectrophotometrically read at 570 nm. The wells filled with SDB supplemented with 2% glucose were used as a blank. Each isolate was tested in triplicate. To determine the biofilm formation capacity of yeasts, the cut-off optical density (ODc) was defined as the sum of the mean OD value for the blank and three times the standard deviation. The intensity of biofilm formation was categorized as follows: nonbiofilm producer (OD < ODc), weak biofilm producer (ODc < OD < 2 × ODc), moderate biofilm producer (2 × ODc < OD ≤ 4 × ODc), and strong biofilm producer (OD > 4 × ODc).

### 2.4. Production of Phospholipase and Proteinase Assays

The production of phospholipase and proteinase was detected using the method described by Lahkar et al. with minor modifications [22]. For the production of phospholipase, 10 *μ*L of yeast inoculum (1.5 × 10^4^ CFU/mL) was transferred onto egg yolk plates and incubated at 37°C for 2 days. An opaque zone around the yeast colony was characterized as the production of phospholipase (Pz) and expressed as Pz = colony diameter/(colony diameter + precipitation zone). The phospholipase was categorized as strong when Pz ≤ 0.63, moderate when 0.64 ≤ Pz ≤ 0.99, and no activity when Pz = 1 [23].

For the production of proteinase, 10 *μ*L of yeast inoculum (1.5 × 10^4^ CFU/mL) was transferred onto agar plates containing bovine serum albumin and incubated at 37°C for 2 days. The production of proteinase (Prz) was observed by a white dense zone of hydrolysis around yeast colonies. Prz was calculated and interpreted as described above for the production of phospholipase. The experiment was performed in triplicate.

### 2.5. Determination of Minimum Inhibitory Concentrations (MICs) and Minimum Fungicidal Concentrations (MFCs)

The antifungal activity was determined by the broth microdilution method [24]. In brief, serial twofold dilutions of natural products and antifungals were made using SDB at a total volume of 100 *μ*L per well in 96-well microplates. Then, 100 *μ*L of fungal inoculum (1.5 × 10^4^ CFU/mL) was added to each well, and the plates were incubated at 37°C for 48 h. The final concentrations of natural products and antifungals ranged from 0.5 to 1024 *μ*g/mL and from 0.125 to 256 *μ*g/mL, respectively. The MIC endpoint is the lowest concentration of natural product or antifungal where no growth was observed in the microplate. For MFC determination, 50 *μ*L of the solution from the wells that showed no visible fungal growth was cultured on SDA plates. Then, the plate was incubated at 37°C for 48 h. The MFC was recorded as the lowest concentration that killed all yeast with no visible viable colonies on an agar plate. The experiment was performed in triplicate and repeated three times. The antifungal activity of natural products was considered as follows: most active (MIC ≤ 10 *μ*g/mL), active (MIC ≤ 25 *μ*g/mL), moderate (25 < MIC ≤ 100 *μ*g/mL), and inactive (MIC > 100 *μ*g/mL) [25]. The epidemiological cut-off values of antifungals described by the Clinical Laboratory Standard Institute were used for *C. neoformans*. For fluconazole, yeast with MIC ≤ 8 *μ*g/mL was considered susceptible while yeast with MIC ≥ 64 *μ*g/mL was considered resistant. For amphotericin B, the MIC value ≤ 1 *μ*g/mL indicated that the yeast was susceptible, while MIC value > 4 *μ*g/mL indicated that the yeast was resistant [26].

### 2.6. Biofilm Inhibition Assay

The activities of natural products and antifungals against the biofilm formation of *C. neoformans* were determined by the microtiter plate method as previously described by Kuaté et al. [14] with slight modifications. In brief, 100 *μ*L of serially double diluted solution concentrations from 0.5 to 1024 *μ*g/mL for natural products or antifungals were added into the wells of the microplate. Then, 100 *μ*L of yeast inoculum (1.5 × 10^6^ CFU/mL) was added to each well, and the microplate was incubated at 37°C for 24 h. After incubation, the planktonic cells were removed by aspiration of the medium and the wells were washed three times with 200 *μ*L of sterile PBS. Then, the microplate was treated as described above for biofilm formation. Untreated wells were used as a positive control while wells containing SDB supplemented with 2% glucose were used as a blank. The percentage of biofilm inhibition of each compound was calculated using the formula (1 − (OD_570_ test − OD_570_ blank)/(OD_570_ control − OD_570_ blank)) × 100. The minimum biofilm inhibitory concentration (MBIC) was defined as the lowest concentration of natural products or antifungals that reduced 100% of the biofilm biomass.

### 2.7. Biofilm Eradication Assay

The capacity of natural products and antifungals to eliminate mature biofilms was determined by using the method described by Kuaté et al. [14]. In brief, after biofilm formation as described above in the biofilm formation assay, the microplate was washed thrice with PBS to remove the nonadherent cells. Then, each well of the microplate was filled with 200 *μ*L of the serial two dilutions at concentrations ranging from 0.5 to 1024 *μ*g/mL, and the plate was incubated at 37°C for 24 h. After incubation, the microplate was treated as described above and the minimum biofilm eradication concentration (MBEC) was recorded as the lowest concentration of natural products or antifungals that eradicated 100% of the preformed biofilm. The test was performed in triplicate and repeated three times.

### 2.8. Statistical Analysis

Statistical analyses were performed using GraphPad Prism version 8.0. The correlation between biofilm formation and phospholipase/proteinase production was determined by Pearson's correlation coefficient (*r*). There is a perfect correlation when *r* = 1, no correlation when *r* = 0, and a negative correlation when *r* = −1. The significance level was set at *p* < 0.05.

## 3. Results

### 3.1. Virulence Factors

Table 1 shows the virulence factors in *C. neoformans*. For biofilm formation, all isolates were able to form biofilms. Based on the cut-off value of ODc = 0.121, four isolates of *C. neoformans* (Cn46, Cn91, Cn96, and Cn118) were classified as strong biofilm producers with OD values ranging from 0.523 ± 0.045 to 0.899 ± 0.057, while the four other isolates of *C. neoformans* (Cn47, Cn158, Cn169, and Cn173) were classified as moderate biofilm producers with OD values ranging from 0.292 ± 0.05 to 0.45 ± 0.018.

All isolates of *C. neoformans* showed strong production of phospholipase except the Cn46 and Cn47 isolates. Strong production of proteinase was observed in *C. neoformans* isolates except for the Cn47, Cn118, Cn169, and Cn173 isolates.

### 3.2. Correlation between Biofilm Formation and Extracellular Enzymes

Correlation analysis results showed a negative correlation between biofilm formation and the following extracellular enzymes: phospholipase (*r* = −0.156, *p*=0.738) and proteinase (*r* = −0.695, *p*=0.085) (Table 2).

### 3.3. Antifungal and Antibiofilm Activities

Table 3 shows the antifungal and antibiofilm potencies of natural products against *C. neoformans*. For antifungal activity, the most active natural product tested in this study was plumbagin with MIC values below 25 *μ*g/mL. Thymol showed moderate antifungal activity against all isolates of *C. neoformans* with MIC values below 100 *μ*g/mL. However, eugenol, piperine, curcumin, and gallic acid were inactive against the majority of *C. neoformans* isolates with MIC values greater than 100 *μ*g/mL. The MIC values of eugenol and piperine ranged from 32 to 256 *μ*g/mL, while the MIC values of curcumin and gallic acid ranged from 64 to 256 *μ*g/mL and 64 to 512 *μ*g/mL, respectively. According to the epidemiological cut-off values of antifungals, all *C. neoformans* isolates were resistant to amphotericin B and fluconazole.

For antibiofilm activity, the MBIC and MBEC values of the natural products and antifungals as well as the MBIC/MIC and MBEC/MBC were determined. The MBIC/MIC and MBEC/MBC ratios demonstrate the increased resistance in yeast living in biofilms compared to their planktonic cells. Plumbagin showed the highest antibiofilm activity with MBIC and MBEC values ranging from 4 to 16 *μ*g/mL and 32 to 256 *μ*g/mL, respectively. Thymol and eugenol exhibited antibiofilm activity, with MBIC values ranging from 32 to 512 *μ*g/mL, whereas the MBIC values of curcumin and gallic acid ranged from 128 to 512 *μ*g/mL. The mature biofilms of *C. neoformans* isolates were destroyed by thymol, eugenol, piperine, and gallic acid with MBEC values ranging from 512 to 1024 *μ*g/mL. The MBIC values of amphotericin B and fluconazole varied from 64 to 128 *μ*g/mL and 16 to 256 *μ*g/mL, respectively, while their MBEC values ranged from 256 to 512 *μ*g/mL. The MBIC/MIC and MBEC/MFC ratios of natural products (1 to 16 and 1 to 8, respectively) were higher than those obtained with antifungals (MBIC/MIC and MBEC/MFC ratios ranged from 0.25 to 2 and 1 to 4, respectively).

## 4. Discussion

In recent years, the emergence of antifungal drug resistance in *C. neoformans* has increased rapidly [27]. Hence, the morbidity and mortality rates due to cryptococcosis caused by *C. neoformans* are increasing continuously. Therefore, new therapeutic strategies need to be explored [11]. In the present study, *C. neoformans* virulence factors such as biofilm formation and the production of phospholipase and proteinase were studied. The different levels of biofilm formation observed in *C. neoformans* isolates could be attributed to the different gene expression levels involved in biofilm formation in these isolates. In particular, a strong biofilm formation was reported in *C. neoformans* isolates [28]. The strong production of phospholipase and proteinase observed in *C. neoformans* isolates would reflect their more virulent character compared to the isolates of *C. neoformans* that produce moderate or weak virulence factors. Our results are similar to those reported earlier [29].

In addition, in the present study, the antifungal activity of thymol, curcumin, piperine, gallic acid, eugenol, and plumbagin was investigated. These natural products showed varying degrees of antifungal activity against *C. neoformans* isolates that could be attributed to their different mechanisms of action. In fact, thymol displayed an antifungal activity against *C. neoformans* by reducing the expression levels of calcium transporter genes in a calcineurin-dependent manner, and the expression of ergosterol biosynthesis genes in a high-osmolarity glycerol (HOG) mitogen-activated protein kinase (MAPK) pathway that responds to a variety of stimuli [30]. Curcumin can bind to the ergosterol present in the membrane, which leads to fungal cell disruption and a loss of intracellular content [31]. Gallic acid and eugenol decreased the activity of sterol 14*α* demethylase P450 (CYP51) and squalene epoxidase, resulting in the inhibition of ergosterol biosynthesis [32, 33]. Plumbagin disrupts the cell membrane integrity and reduces the metabolic activity of *C. neoformans* [15]. Few studies have reported similar results of the antifungal activity of the studied natural products against *C. neoformans.* Kumari et al. reported the antifungal activity of thymol at a concentration of 16 *μ*g/mL against *C. neoformans* [34]. Hassanpour et al. showed that eugenol at concentrations of 250 *μ*g/mL and 500 *μ*g/mL displayed 90% and 100% growth inhibition in *C. neoformans*, respectively [35]. Qian et al. reported the antifungal activity of plumbagin with an MIC value of 8 *μ*g/mL against *C. neoformans* [15].

The antibiofilm activity of these natural products was also investigated. It was observed that the MBIC values of natural products and antifungals were up to 16 times and 2 times higher than their MIC values, respectively. In addition, the MBEC values of natural products and antifungals were up to 8 times and 4 times higher than their MFC values, respectively. These results showed that the biofilm was more resistant to antimicrobial agents than planktonic cells, and this could be explained by the limited diffusion of antimicrobial agents through the biofilm matrix or the lower growth rate of cells in the biofilm [6, 36]. These results corroborated those reported earlier [14, 24]. Kumari et al. reported the antibiofilm activity of thymol and eugenol against *C. neoformans* [34]. In addition, Qian et al. reported the antibiofilm activity of plumbagin with MBIC and MBEC values of 64 *μ*g/mL and 128 *μ*g/mL against *C. neoformans,* respectively [15].

## 5. Conclusion

The natural products studied in this study were found to possess antifungal and antibiofilm activities against *C. neoformans.* In particular, plumbagin was found to be the most active against planktonic cells and biofilms of *C. neoformans.* Therefore, new studies are required to investigate the efficacy of combination therapies of plumbagin with antifungals, which could lead to the development of novel therapies against recalcitrant infections caused by *C. neoformans.*

## Figures and Tables

**Table 1:** Virulence factors in *C. neoformans* isolates.

Isolates	Virulence factors		
	Biofilm formation (OD_570 nm_)	Phospholipase (Pz value)	Proteinase (Prz value)
Cn46	0.523 ± 0.045	0.71	0.62
Cn47	0.312 ± 0.007	0.75	0.88
Cn91	0.899 ± 0.057	0.50	0.41
Cn96	0.562 ± 0.019	0.59	0.56
Cn118	0.704 ± 0.060	0.37	0.67
Cn158	0.456 ± 0.018	0.44	0.52
Cn169	0.292 ± 0.070	0.33	0.67
Cn173	0.363 ± 0.031	0.59	0.69

OD = optical density; Cn, *C. neoformans*; Pz, production of phospholipase; Prz, production of proteinase.

**Table 2:** Correlation between biofilm formation and phospholipase/proteinase production.

	*Biofilm formation*	
	*r*	*p* value
Phospholipase	−0.156	0.738
Proteinase	−0.695	0.085

*r*, Pearson's correlation coefficient.

**Table 3:** Antifungal and antibiofilm activities of natural products against *C. neoformans* isolates.

Isolates		*Natural products*						*Antifungals*	
		Thy	Plu	Eug	Pip	Cur	Agl	AmB	Flu
Cn46	MIC	64	8	128	32	64	128	128	32
	MFC	256	32	512	128	256	512	256	128
	MBIC	128	16	512	64	256	512	128	16
	MBIC/MIC	2	2	4	2	1	4	1	0.5
	MBEC	512	128	1024	1024	1024	1024	512	256
	MBEC/MFC	2	4	2	4	4	2	2	2

Cn47	MIC	64	4	256	128	128	256	256	256
	MFC	64	16	256	256	256	1024	256	512
	MBIC	256	4	512	256	512	128	64	128
	MBIC/MIC	4	1	2	1	4	0.5	0.25	0.5
	MBEC	512	128	512	512	1024	1024	256	512
	MBEC/MFC	8	8	2	2	4	1	1	1

Cn91	MIC	32	8	64	256	128	128	64	32
	MFC	128	32	256	1024	512	512	128	128
	MBIC	128	16	128	512	256	256	128	32
	MBIC/MIC	4	2	2	2	2	2	2	1
	MBEC	1024	256	1024	1024	1024	512	256	256
	MBEC/MFC	8	8	4	1	2	1	2	2

Cn96	MIC	32	8	128	128	256	256	256	32
	MFC	128	16	512	512	512	256	512	64
	MBIC	64	8	512	512	512	512	128	64
	MBIC/MIC	2	1	4	4	2	2	0.5	0.5
	MBEC	1024	128	1024	1024	1024	1024	512	512
	MBEC/MFC	8	8	2	8	2	4	1	8

Cn118	MIC	32	4	128	256	256	64	256	256
	MFC	256	32	512	256	512	256	256	512
	MBIC	32	16	256	256	512	128	128	128
	MBIC/MIC	1	2	2	1	1	2	0.5	0.5
	MBEC	512	128	1024	256	1024	512	512	512
	MBEC/MFC	2	4	2	1	2	2	2	1

Cn158	MIC	8	4	32	64	128	512	128	64
	MFC	32	4	128	256	512	1024	512	256
	MBIC	8	4	32	128	256	512	64	128
	MBIC/MIC	0.25	1	1	2	2	1	0.5	2
	MBEC	512	32	1024	1024	1024	1024	512	256
	MBEC/MFC	16	8	8	4	2	1	1	1

Cn169	MIC	64	8	128	128	128	256	64	256
	MFC	64	8	256	256	256	512	128	512
	MBIC	64	8	64	256	256	512	128	128
	MBIC/MIC	1	1	0.5	2	2	2	2	0.5
	MBEC	256	32	512	1024	512	1024	512	512
	MBEC/MFC	4	4	2	4	2	2	4	1

Cn173	MIC	32	16	256	256	256	128	256	256
	MFC	128	32	512	256	512	512	256	512
	MBIC	32	4	512	512	128	128	128	256
	MBIC/MIC	0.5	0.25	2	2	0.5	1	0.5	1
	MBEC	1024	256	1024	1024	1024	512	512	512
	MBEC/MFC	8	8	2	4	2	1	2	1

MIC: minimum inhibitory concentration (*μ*g/mL); MFC: minimum fungicidal concentration (*μ*g/mL); MBIC: minimum biofilm inhibitory concentration (*μ*g/mL); MBEC: minimum biofilm eradication concentration (*μ*g/mL); Thy: thymol; Plu: plumbagin; Eug: eugenol; Pip: piperine; Cur: curcumin; Agl: gallic acid; AmB: amphotericin B; Flu: fluconazole; Cn: *C. neoformans.*

## Data Availability

The data used to support the findings of the study can be obtained from the corresponding author upon request.
